# USP22-dependent *HSP90AB1* expression promotes resistance to HSP90 inhibition in mammary and colorectal cancer

**DOI:** 10.1038/s41419-019-2141-9

**Published:** 2019-12-04

**Authors:** Robyn Laura Kosinsky, Marlena Helms, Maria Zerche, Luisa Wohn, Anna Dyas, Evangelos Prokakis, Zahra Basir Kazerouni, Upasana Bedi, Florian Wegwitz, Steven A. Johnsen

**Affiliations:** 1 Department of General, Visceral and Pediatric Surgery, University Medical Center Göttingen, Justus-von-Liebig-Weg 11, 37077 Göttingen, Germany; 2 Gene Regulatory Mechanisms and Molecular Epigenetics Lab, Division of Gastroenterology and Hepatology, Mayo Clinic, 200 First St SW, Rochester, MN USA

**Keywords:** Cancer epigenetics, Targeted therapies

## Abstract

As a member of the 11-gene “death-from-cancer” gene expression signature, overexpression of the *Ubiquitin-Specific Protease 22* (*USP22*) was associated with poor prognosis in various human malignancies. To investigate the function of USP22 in cancer development and progression, we sought to detect common USP22-dependent molecular mechanisms in human colorectal and breast cancer cell lines. We performed mRNA-seq to compare gene expression profiles of various colorectal (SW837, SW480, HCT116) and mammary (HCC1954 and MCF10A) cell lines upon siRNA-mediated knockdown of USP22. Intriguingly, while USP22 depletion had highly heterogeneous effects across the cell lines, all cell lines displayed a common reduction in the expression of *Heat Shock Protein 90 Alpha Family Class B Member 1* (*HSP90AB1*). The downregulation of HSP90AB1 was confirmed at the protein level in these cell lines as well as in colorectal and mammary tumors in mice with tissue-specific *Usp22* deletions. Mechanistically, we detected a significant reduction of H3K9ac on the *HSP90AB1* gene in USP22-deficient cells. Interestingly, USP22-deficient cells displayed a high dependence on *HSP90AB1* expression and diminishing HSP90 activity further using the HSP90 inhibitor Ganetespib resulted in increased therapeutic vulnerability in both colorectal and breast cancer cells in vitro. Accordingly, subcutaneously transplanted CRC cells deficient in *USP22* expression displayed increased sensitivity towards Ganetespib treatment in vivo. Together, we discovered that *HSP90AB1* is USP22-dependent and that cooperative targeting of USP22 and HSP90 may provide an effective approach to the treatment of colorectal and breast cancer.

## Introduction

Despite improved diagnostic and therapeutic approaches, malignant diseases remain a major health burden worldwide. In fact, studies indicate that cancer has replaced cardiovascular disorders as the leading cause of death in several countries^1^. This emphasizes the need to improve the current understanding of the development, progression and recurrence of malignant diseases. Interestingly, the overexpression of a specific group of genes identified as the so-called 11-gene “death-from-cancer” expression signature was associated with particularly poor patient survival, distant metastasis and high recurrence rates^2,3^. The *Ubiquitin-Specific Protease 22* (*USP22*) is a member of this signature and has been described in numerous studies to be overexpressed in various human malignancies^4^. These studies were mainly based on immunohistochemical analyses or evaluations of mRNA. USP22, together with Ataxin 7 (ATXN7), Ataxin 7-Like 3 (ATXN7L3) and *Enhancer of Yellow 2* Homolog (ENY2), forms the deubiquitinating module (DUBm) of the SAGA (Spt-Ada-Gcn5 Acetyltransferase) complex. In addition to the DUBm, the SAGA transcriptional cofactor complex contains the histone acetyltransferase (HAT) General Control Nonderepressible 5 (GCN5), which promotes transcription via acetylation of lysine 9 of histone 3 (H3K9ac)^5^. Interestingly, in contrast to the prevailing view that USP22 is a universal oncogene, our recent results demonstrated a context-dependent tumor suppressor function of USP22 in colorectal cancer whereby loss of *USP22* expression resulted in decreased SAGA-mediated H3K9ac on the *PRKAA2* gene (which encodes the AMP-activated protein kinase-2) and a concomitant downregulation of its expression, thereby leading to activation of the mTOR signaling pathway^6^. Importantly, while loss of *USP22* expression resulted in increased tumor growth and aggressiveness, activation of the mTOR pathway resulted in a synthetic vulnerability of USP22-deficient colorectal cancer cells to mTOR inhibitor treatment.

Mechanistically, USP22 was reported to deubiquitinate the core histone H2B at lysine 120^7^. The loss of this monoubiquitination (H2Bub1) has been associated with advanced tumor grade and poor patient survival in colorectal (CRC)^8^ and breast cancer^9^ and, therefore, H2Bub1 has been considered as a tumor-suppressive epigenetic mark. Apart from its function in deubiquitinating H2B, USP22 was also reported to deubiquitinate and thereby stabilize several key oncogenic proteins including MYC^7^ and Sirtuin 1 (SIRT1)^10^. Based on its function in deubiquitinating H2B and oncogenic proteins, increased USP22 levels were reported to accelerate colorectal^11–14^ and breast cancer development and progression^15,16^. Thus, USP22 has been proposed as an attractive therapeutic target in malignant diseases and, indeed, there is ongoing research to generate and optimize USP22 inhibitors^4^, although caution must be used given our findings of the context-dependent function of USP22 in cancer.

In this study, we aimed to investigate the function of USP22 in colorectal and breast cancer and to detect common USP22-dependent molecular mechanisms which may be exploited for cancer treatment. For this purpose, we performed next-generation sequencing in several human cell lines and employed genetic tumor mouse models with intestine- and mammary-specific deletions of *Usp22*. Finally, after identifying *HSP90AB1* as a novel USP22-dependent target gene, we evaluated the therapeutic targetability of USP22-deficient tumor cells in vitro and in vivo.

## Materials and methods

### Cell culture and siRNA-mediated knockdowns

Human cell lines were grown in their respective growth media supplemented with 10% fetal bovine serum, 100 units/ml penicillin and 100 μg/ml streptomycin at 37 °C and 5% CO_2_ (SW837: DMEM/F-12, SW480: RPMI Glutamax, HCT116: McCoy’s, HCC1954: RPMI Glutamax, MCF10A: DMEM/F-12 supplemented with 5% horse serum, 0.5 μg/ml hydrocortisone, 10 μg/ml Insulin, 20 ng/ml human epithelial growth factor, 0.1 μg/ml cholera toxin). siRNA (GE Dharmacon siGENOME; Table S1) transfections using a non-targeting control (NT5) or targeting USP22 or GCN5 were performed using Lipofectamine® RNAiMAX (Invitrogen) according to the manufacturer’s instructions. To test the effect of HSP90 inhibition cells were treated with the indicated concentrations of Ganetespib (Selleckchem) 24 h after siRNA-mediated knockdown for an additional 48 h. As a negative control, DMSO was added to the cells.

### CRISPR/Cas9-mediated deletion of *USP22*

The HCT116 colorectal cancer cell lines harboring a permanent deletion of the *USP22* gene were described previously^6^. Briefly, two single guide RNAs (sgRNAs) targeting *USP22* (sgRNA1: 5′-CACCGGTGTTTGGCAGCTCATGCCC-3′, sgRNA2: 5′-CACCGTTAGAGAGACCTGGCGGTGG-3′) were cloned into the pSpCas9(BB)-2A-GFP (PX458, Addgene) vector containing Cas9 and GFP sequences. Single highly fluorescent cells were sorted into 96-well plates using fluorescence activated cell sorting (FACS) and single cell clones were expanded and the loss of USP22 was confirmed by western blot and qRT-PCR. To avoid potential off-target effects, two HCT116 *USP22*^−/−^ single cell clones were characterized and one non-targeted *USP22*^+/+^ single cell clone as well as the HCT116 parental cell line have been used as controls. Unless indicated otherwise, results obtained from one representative clone are shown.

### Proliferation assays

To determine cell proliferation, 2000 HCT116 and 3000 HCC1954 cells were seeded onto 96-well assay plates (Corning Life Sciences) and confluence was measured daily using a Celigo® S Adherent Cell Cytometer (Nexcelom Bioscience LLC). The effect of Ganetespib treatment on cell confluence was tested by seeding 20,000 HCT116 and 30,000 HCC1954 cells per well of a 24-well plate and staining cells using 1% (w/v) crystal violet in 20% ethanol after 48 h of HSP90i treatment.

### Western blotting and qRT-PCR

Protein isolation, western blot analysis, RNA extraction, reverse transcription, and qRT-PCR were performed as previously described^17^. The primary antibodies for western blot and primers for qRT-PCR are listed in Tables S2 and S3, respectively.

### Chromatin immunoprecipitation

Chromatin immunoprecipitation (ChIP) was performed to assess H3K9ac occupancy on the *HSP90AB1* gene. HCT116 *USP22*^+/+^ and *USP22*^−/−^ cells were fixed in 1% formaldehyde in PBS for 20 min and quenched with 125 mM glycine for 5 min. After washing cells twice with ice-cold PBS, they were scraped and washed with Nelson Buffer (150 mM NaCl, 20 mM EDTA (pH 8.0), 50 mM Tris/HCl (pH 7.5), 0.5% (v/v) NP-40, 1% (v/v) Triton-X-100, 20 mM NaF and protease inhibitors). The pellets were resuspended in Lysis Buffer (20 mM EDTA, 150 mM NaCl, 1% (v/v) NP-40, 0.1% (w/v) SDS, 0.5% (w/v) sodium deoxycholate, 20 mM NaF, 50 mM Tris/HCl (pH 8.0) and protease inhibitors) and sonicated for 15 cycles (30 s on/off). After preclearing with 50% Sepharose 4B (GE Healthcare) for 1 h, samples were incubated with antibodies at 4 °C overnight (H3K9ac: C15410004, Diagenode; IgG: C15410206, Diagenode). Sepharose beads with Protein A (GE Healthcare) were added and incubated at 4 °C for 2 h. Samples were washed twice with Wash Buffer (20 mM EDTA, 500 mM LiCl, 1% (v/v) NP-40, 20 mM NaF, 1% (w/v) sodium deoxycholate, 100 mM Tris/HCl (pH 8.5)) and twice with TE buffer. Next, immune complexes were incubated with RNAse A (Qiagen) in 10 mM Tris/HCl (pH 8.0) at 37 °C for 30 min and de-crosslinked in 20 mM EDTA, 2% (w/v) SDS, 100 mM Tris/HCl (pH 8) and 20 μg Proteinase K at 65 °C overnight. DNA was extracted with Roti® phenol/chloroform/isoamylalcohol (Roth) and precipitated with ethanol. Input samples were used as controls and primer sequences for subsequent qRT-PCR are listed in Table S4.

### Library preparation and next-generation sequencing

To evaluate transcriptome-wide effects of USP22 loss, mRNA-seq and subsequent analysis was performed. SW837, SW480, HCT116, MCF10A and HCC1954 cells were transfected with control or USP22 siRNAs (*n* = 3–4 per condition). RNA integrity was verified using agarose gel electrophoresis and libraries were prepared using TruSeq RNA Library Preparation Kit v2 (Illumina®) or NEXTflex Rapid Directional RNA-Seq Kit (Biooscientific). Sequencing was performed at the Transcriptome and Genome Analysis Laboratory (TAL, Göttingen) using HiSeq 2000 or 4000 (Illumina®). All mRNA-seq data have been deposited at ArrayExpress (http://www.ebi.ac.uk/arrayexpress, accession numbers: E-MTAB-7393 (HCT116)^6^, E-MTAB-8214 (SW480), E-MTAB-8215 (SW837), E-MTAB-8247 (MCF10A), E-MTAB-8256 (HCC1954)). Data analysis and Gene Set Enrichment Analysis (GSEA;^18^), were performed as previously described^17,19^.

### Generation of mice and genotyping

All animal work was performed according to institutional regulations for care and use of laboratory animals and approved by the Lower Saxony State Office for Consumer Protection and Food Safety (LAVES; registration numbers: G15/2039, G15/1754 and G11/540). *Usp22*^flox^ mice were generated as described previously^6^ by removing lacZ and neomycin resistance loci from the *Usp22*^tm1a(KOMP)Wtsi^ C57BL6 mouse line generated from embryonic stem cells obtained from the University of California-Davis Knockout Mouse Project Repository^20^ by FLP-mediated excision^21^. *Usp22*^flox^ mice were crossed with MMTV-Cre (a kind gift from L. Henninghausen, National Institutes of Health, USA) and Tg(MMTV-ErbB2)NK1Mul/J (The Jackson Laboratory) animals (FVB/N background) to achieve a mammary-specific *Usp22* knockout and to promote tumorigenesis, respectively. Moreover, *Usp22*^flox^ mice were crossed with Villin-Cre^ERT2^ and *Apc*^1638N^ mouse lines (C57BL/6N background) to achieve an intestinal knockout and to investigate its role in tumorigenesis^6^. The Apc^1638N/+^ mouse line was a kind gift from F. Bosman (Erasmus University Medical Center Rotterdam, The Netherlands). Multiple replicates (*n* = 3–6) were utilized in order to ensure reproducibility of findings.

### Tamoxifen treatment

4-week-old Villin-Cre^ERT2^, *Usp22*^flox^, and Villin-Cre^ERT2^, *Apc*^1638N/+^, *Usp22*^flox^ mice were injected with a total dose of 1 mg Tamoxifen (Sigma-Aldrich) per day for five consecutive days to induce an intestinal knockout of *Usp22*. Tamoxifen (5% w/v) was dissolved in 100% ethanol and mixed 1:10 with autoclaved sunflower oil right before intraperitoneal injection.

### Xenograft experiments

Immunodeficient SCID mice were housed in a controlled, germ-free environment. Animals were randomized into treatment groups and anesthetized by isoflurane inhalation (2–3%, Forene) and HCT116 *USP22*^+/+^ and *USP22*^−/−^ cells (1.0 × 10^6^ cells in Corning® Matrigel®) were injected subcutaneously into the left and right flank, respectively, using a 0.3 ml Micro-Fine syringe (BD Bioscience). The bodyweight, as well as the size of the growing tumors, were monitored daily. After the tumors reached a volume of approximately 100 mm³, 25 mg/kg Ganetespib (AdooQ) or vehicle solution (10% DMSO (v/v), 20% (v/v) pre-warmed Cremophor RH40 (55 °C; Sigma-Aldrich), 5% (w/v) dextrose) was injected i.v. for three consecutive days and after 4 days break, for 3 further days. Experiments were performed with six replicates per group in order to assure reproducibility. Experiments were performed blinded during tumor size measurement and subsequent immunohistochemical staining.

### H&E staining and immunohistochemistry (IHC)

Histological staining was performed as described previously^20^. Briefly, 5 µm tissue sections were de-paraffinized in xylol for 20 min, rehydrated using decreasing ethanol concentrations (100%, 90%, 70%) for 5 min each and washed with water. For H&E staining, slides were stained in Mayer’s hematoxylin solution (Roth) for 5 min and counterstained with eosin for 5–10 min. For IHC, sections were boiled in 10 mM citric acid buffer for 15 min and incubated with 5% H_2_O_2_ in PBS. Upon blocking with 10% fetal bovine serum (FBS) in PBS, sections were incubated with primary antibodies (Table S2) diluted in 5% FBS in PBS in a humid chamber at 4 °C overnight. Biotinylated secondary antibodies (1:200; GE Healthcare) and ExtrAvidin-Peroxidase (1:1,000; Sigma-Aldrich) diluted in PBS were added each for 1 h. Staining was developed using 3,3′-diaminobenzidin-tetrahydrochloride (DAB; Roth) and counterstaining was performed using hematoxylin. Slides were dehydrated, incubated in xylol and mounted. To quantify HSP90AB1 IHC staining intensity we utilized the “color deconvolution” tool in FIJI to separate the DAB signal from the hematoxylin/background signals. The maximum intensity was divided by the minimum intensity in 4–6 images per staining per mouse.

### Statistical analyses

All graphs have been designed and the area under the curve (AUC) has been calculated with GraphPad Prism version 5.04 (GraphPad Software, Inc.). Statistical analysis was performed using Student’s *t*-test or one-way ANOVA and subsequent Tukey Post hoc test (*α* = 0.05). Variation between different groups that were statistically compared was similar.

## Results

### USP22-deficiency decreases mRNA levels of heat shock factor *HSP90AB1*

**Fig. 1:**
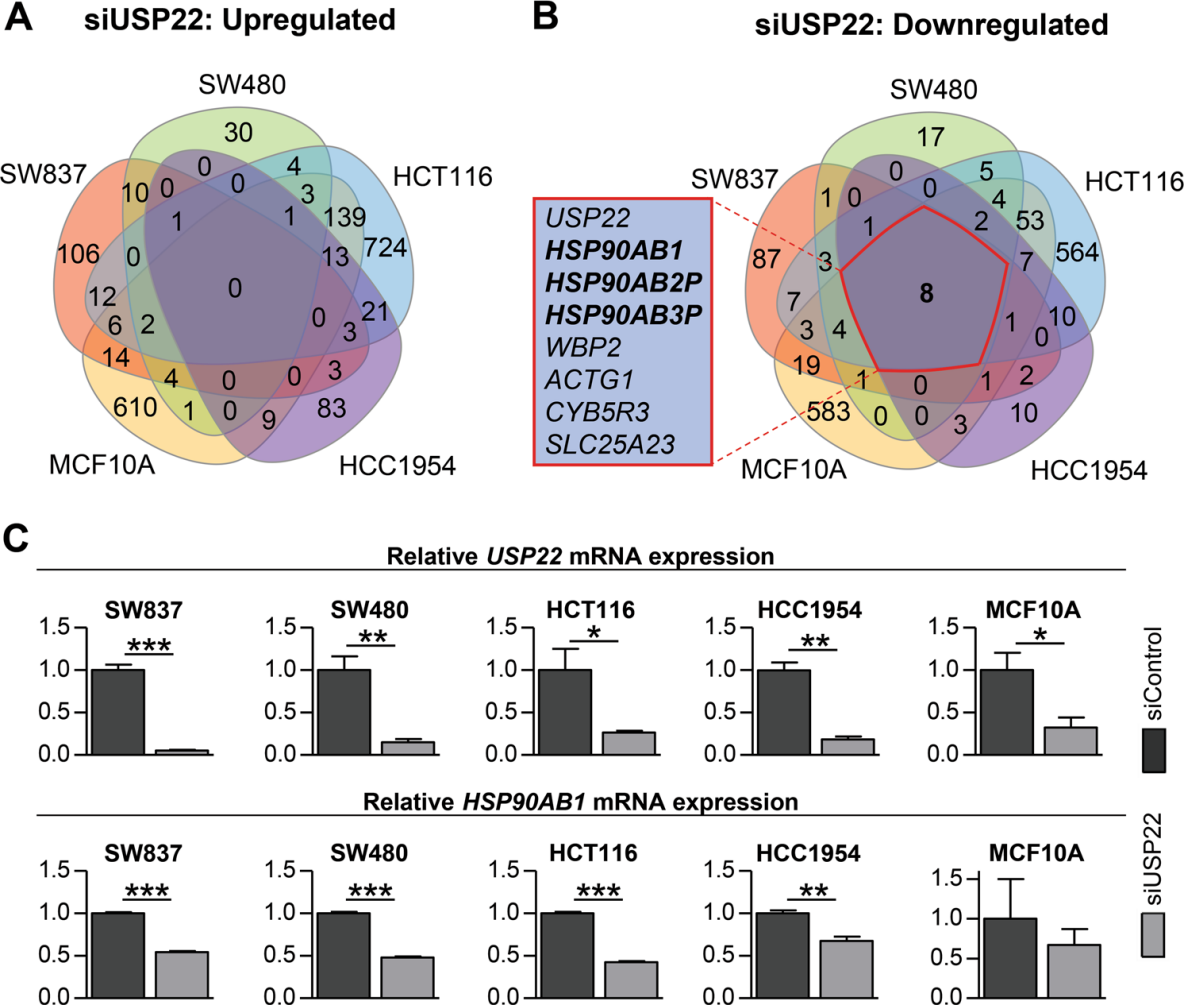
USP22 loss reduces *HSP90AB1* mRNA expression. **a** siRNA-mediated USP22 knockdowns were performed in SW837, SW480, HCT116, MCF10A, and HCC1954 cells (*n* = 3–4). While no overlap was detected in the upregulated genes, **b** all five human cell lines displayed reduced expression of the heat shock factors *HSP90AB1*, *HSP90AB2P*, and *HSP90AB3P*. **c** As verified using qRT-PCR in three independent experiments, the siRNA-mediated depletion of USP22 resulted in reduced *HSP90AB1* mRNA levels. Mean ± SEM, Student’s *t*-test.

While our recent work suggested that USP22 can have context-dependent effects in cancer and have either tumor-promoting or suppressing functions^6^, previous studies have largely reported that overexpression of *USP22* is associated with a more aggressive tumor phenotype. Notably, human tumor gene expression data generated by the TCGA Research Network (http://cancergenome.nih.gov/;^22^) implied that a significant proportion of colorectal cancer (22%) and breast cancer patients (26%) display low *USP22* expression (Fig. S1A). Thus, we sought to obtain a broad overview of the transcriptome-wide consequences of USP22 depletion in various colorectal and mammary cancer cell lines in vitro. Therefore, we performed siRNA-mediated knockdowns of USP22 (siUSP22) and compared these to control knockdowns (siControl) via mRNA-seq analysis in five different human cell lines. These cells originate from rectum adenocarcinoma (SW837), colorectal adenocarcinoma (SW480), colorectal carcinoma (HCT116), non-transformed mammary epithelium (MCF10A) and HER2-positive mammary ductal carcinoma (HCC1954). As expected based on our previous findings, a high degree of heterogeneity was observed in the effects elicited by USP22 depletion in the various cell lines. While no mutually upregulated genes were detected (Fig. 1a), we identified eight mutually downregulated genes including the Heat Shock Protein 90 encoding gene *HSP90AB1* as well as two HSP90 pseudogenes *HSP90AB2P* and *HSP90AB3P* (Fig. 1b). Indeed, the reduction of *HSP90AB1* mRNA levels was confirmed using qRT-PCR in all five cell lines, yet not statistically significant in MCF10A cells (Fig. 1c). Together, upon the transient depletion of USP22 we detected a downregulation of *HSP90AB1* by up to 60% in five human cell lines of various origins.

### USP22-deficiency influences HSP90-dependent signaling

**Fig. 2:**
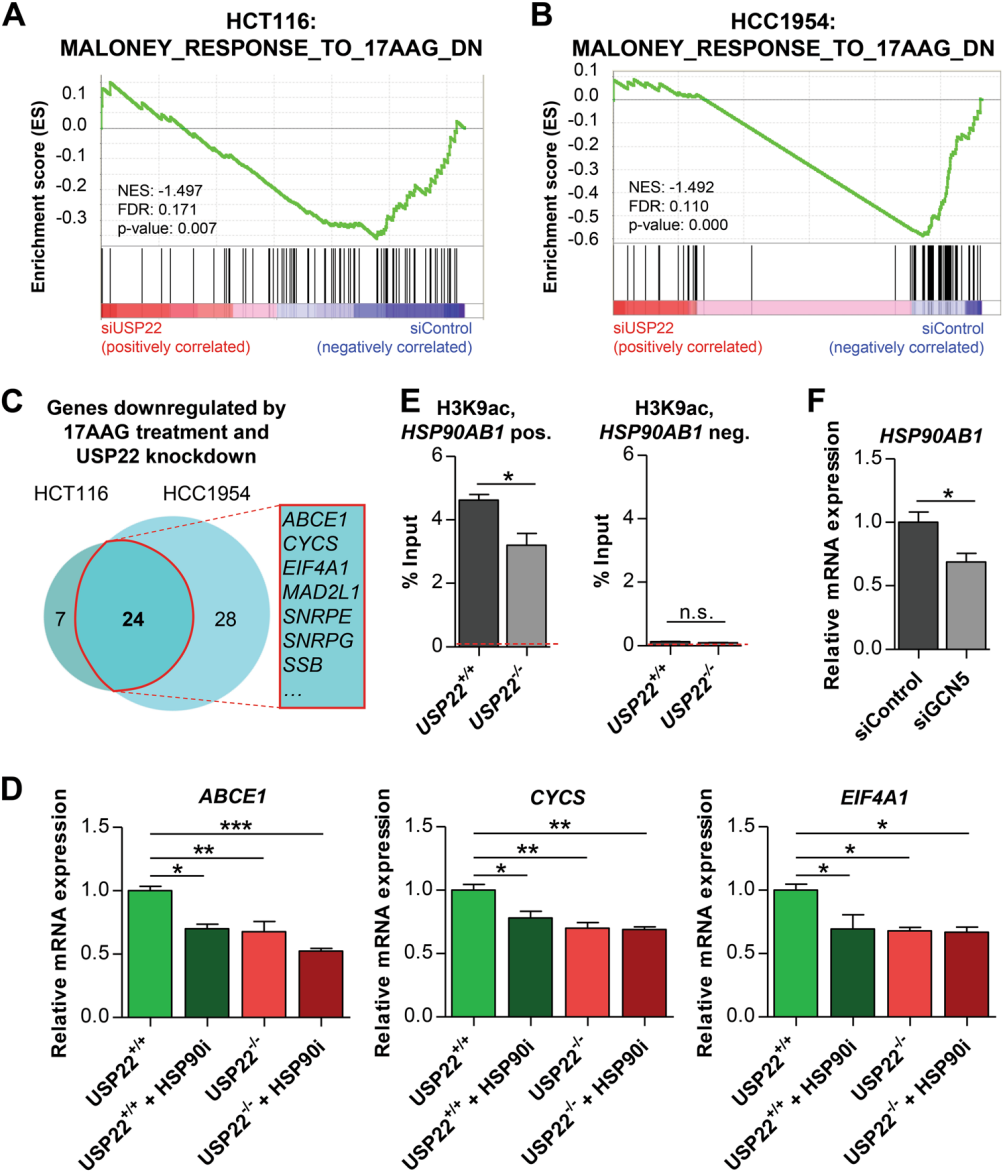
HSP90-dependent signaling is affected by USP22. **a** GSEA indicated that genes, which are downregulated upon 17-AAG treatment are enriched in siUSP22 HCT116 and **b** HCC1954 cells. **c** Venn diagram displaying the overlap of HSP90i- and USP22 knockdown-responsive genes in HCT116 and HCC1954 cells. **d** The dependency of *ABCE1*, *CYCS* and *EIF4A1* on HSP90 as well as *USP22* was verified by treating HCT116 *USP22*^+/+^ and *USP22*^−/−^ cells with the HSP90i Ganetespib or DMSO as a negative control. Mean ± SEM, one-way ANOVA. **e** ChIP-qPCR revealed in two independent experiments that the H3K9ac occupancy on the *HSP90AB1* gene is significantly reduced upon the deletion of *USP22* in HCT116 cells (*n* = 4). An adjacent H3K9ac-negative site was analyzed as a control. The average signal for the negative control IgG is represented by dotted lines. Mean ± SEM, *t*-test. **f** siRNA-mediated silencing of the acetyltransferase GCN5 reduced *HSP90AB1* mRNA levels in HCT116 cells (*n* = 3). Mean ± SEM, *t*-test.

HSP90AB1 functions as a constitutively active molecular chaperone and was found to be overexpressed in various malignant diseases^23^. Therefore, over the last decades, numerous HSP90 inhibitors (HSP90i) have been developed and tested in clinical trials^24^. Interestingly, when elucidating gene expression signatures enriched following USP22 depletion by performing Gene Set Enrichment Analysis (GSEA), while the individual gene expression changes between cell lines were highly variable, we were able to detect an enrichment of genes downregulated upon the treatment with the HSP90i 17-AAG in HCT116 (Fig. 2a) and HCC1954 cells (Fig. 2b). Among these genes were 24 which were mutually regulated in both cell lines. These genes included *ATP-Binding Cassette sub-family E member 1* (*ABCE1)*, *Cytochrome C Somatic* (*CYCS)* and *Eukaryotic Translation Initiation Factor 4A1* (*EIF4A1)* (Fig. 2c). To obtain further mechanistic insights, we utilized HCT116 cells with a permanent CRISPR/Cas9-mediated deletion of the *USP22* gene^6^. Consistent with siRNA-mediated knockdown, these cells displayed a reduction of *HSP90AB1* at the mRNA level (Supplementary Fig. S1B). Next, to confirm the dependency of *ABCE1*, *CYCS* and *EIF4A1* expression on HSP90 as well as USP22, HCT116 wild type and *USP22* knockout cells were treated with the HSP90i Ganetespib. Indeed, Ganetespib treatment or *USP22* deletion resulted in reduced mRNA expression of these genes (Fig. 2d). Since USP22 functions to regulate transcription as a member of the SAGA complex, we next performed ChIP for H3K9ac, a histone modification mediated by the SAGA acetyltransferase GCN5, on the *HSP90AB1* gene in HCT116 cells. Notably, consistent with a direct role of USP22 and the SAGA complex, H3K9ac occupancy on the *HSP90AB1* gene was significantly decreased in *USP22* knockout cells compared to wild type cells (Fig. 2e). As a control, a H3K9ac-negative site on the same gene was examined. Consistent with a general role of SAGA in the regulation of *HSP90AB1* mRNA expression, *HSP90AB1* mRNA levels were also reduced following siRNA-mediated depletion of GCN5 (Fig. 2f, Supplementary Fig. S1C). In summary, in USP22-deficient HCT116 cells, GCN5-mediated H3K9ac occupancy on *HSP90AB1* is reduced, thereby resulting in decreased *HSP90AB1* mRNA expression and down-regulation of HSP90-dependent genes.

### USP22 loss reduces HSP90AB1 protein levels in vitro and in vivo

**Fig. 3:**
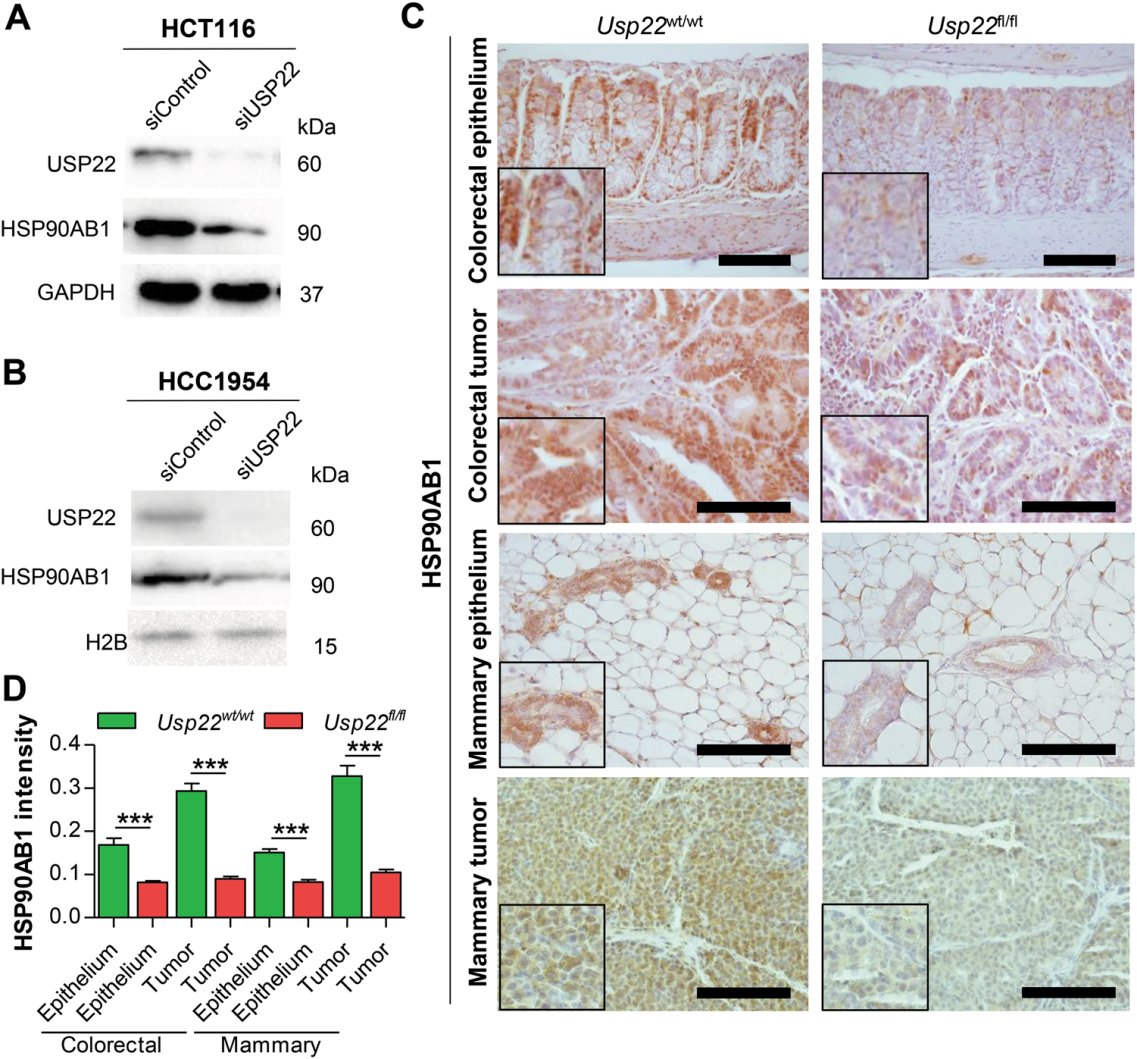
USP22 loss results in reduced HSP90AB1 protein levels. **a** Western blot analysis was performed to demonstrate the reduction of HSP90AB1 protein levels after USP22 knockdown in HCT116 and **b** HCC1954 cells (*n* = 3). **c** Villin-Cre^ERT2^, *Usp22*^flox^ animals were generated to delete *Usp22* specifically in the intestinal epithelium. In comparison, colorectal tumors from *Villin*-Cre^ERT2^, *Apc*^1638N/+^, *Usp22*^flox^ mice and mammary tumors from MMTV-Her2, MMTV-Cre, *Usp22*^flox^ animals were examined. As demonstrated using IHC, *Usp22* deletion resulted in decreased HSP90AB1 staining in both intestinal (*n* = 6) and mammary epithelium (*n* = 3) as well as in colorectal (*n* = 6) and mammary tumors (*n* = 5). Scale bar: 100 µm. **d** HSP90AB1 IHC staining intensity was quantified using FIJI in 4–6 images per staining per mouse. Mean ± SEM, *t*-test.

To verify that the downregulation of *HSP90AB1* mRNA expression results in reduced HSP90AB1 protein levels upon depletion of USP22, we performed western blot analysis in HCT116 and HCC1954 cells. Consistent with the effects observed on mRNA levels, we also observed a decrease in HSP90AB1 protein levels in USP22 knockdown cells (Fig. 3a, b). We next sought to substantiate these findings in vivo via immunohistochemistry for HSP90AB1 in tissue sections from *Usp22*-deficient murine models. For this purpose, we utilized our previously published Villin-Cre^ERT2^, *Usp22*^flox^ mouse model containing an intestine-specific, conditional *Usp22* knockout^6^. Here, normal colorectal epithelium displayed reduced HSP90AB1 staining (Fig. 3c, d). Importantly, tumors derived from Villin-Cre^ERT2^, *Usp22*^flox^ mice containing a mutation in the *Adenomatous Polyposis Coli* (*APC*) tumor suppressor gene (APC^1638N/+^) similarly displayed decreased HSP90AB1 levels compared to the *Usp22* wild type control tumors. Finally, this finding could be further confirmed in normal mammary epithelium and mammary tumors derived from mice containing a MMTV-Cre driven mammary-specific deletion^25^ of *Usp22* in a Her2-driven model of breast cancer. Together, these findings suggest that USP22 is required for high level expression of HSP90AB1 protein levels both in human cancer cell lines in vitro as well as in murine epithelium and tumors in vivo.

### USP22 deficiency sensitizes CRC and breast cancer cells towards HSP90i

**Fig. 4:**
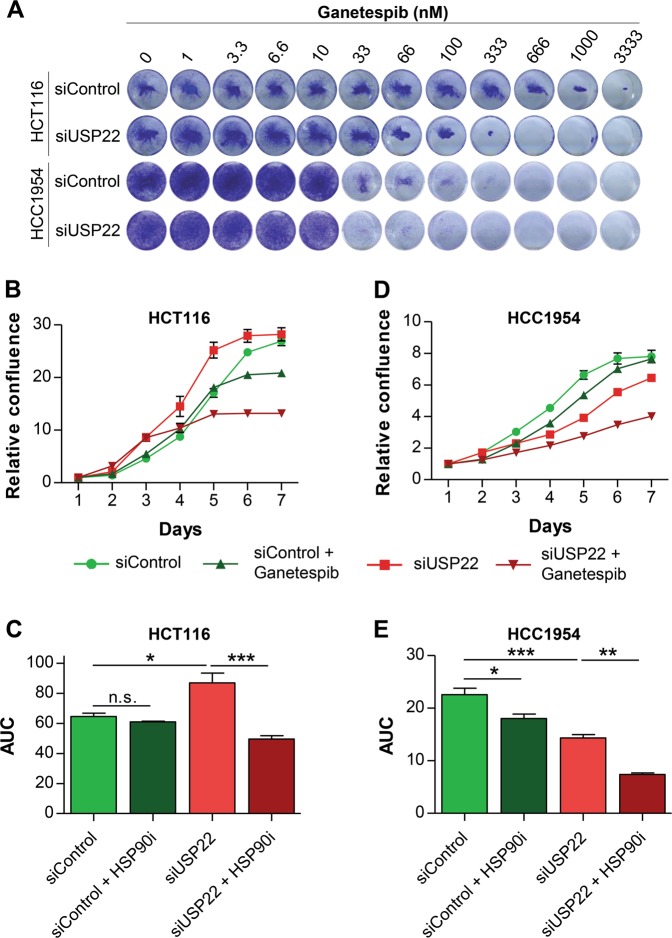
USP22 deficiency sensitizes cancer cells towards HSP90 inhibition. **a** HCT116 and HCC1954 control and USP22-depleted cells were treated with increasing concentrations of the HSP90i Ganetespib for 48 h and visualized using crystal violet. USP22-depleted cells showed increased sensitivity towards Ganetespib. **b** The confluence of USP22 knockdown cells upon Ganetespib treatment was decreased in HCT116 cells as revealed by daily measurements of cell confluence (*n* = 6) as well as by **c** subsequent calculation of the area under the curve (AUC). **d**, **e** This finding was confirmed in HCC1954 cells (*n* = 6). Mean ± SEM, one-way ANOVA.

Based on the finding that USP22 loss reduces HSP90AB1 levels, we hypothesized that the residual HSP90 levels might be essential for the survival and stress-resistance of USP22-depleted cells. Therefore, we further hypothesized that USP22-deficient cells may therefore be particularly sensitive to the inhibition of the remaining HSP90 activity. Therefore, we treated control and USP22-depleted HCT116 and HCC1954 cells with increasing concentrations of Ganetespib for 48 h and visualized cell number via crystal violet staining. Indeed, both cell lines displayed increased sensitivity towards Ganetespib following USP22 knockdown (Fig. 4a). These results were further verified by measuring cellular confluence under the same experimental conditions using a Celigo® S Adherent Cell Cytometer (Fig. 4b–e). Moreover, this effect was not a non-specific effect due to siRNA transfection since the increased sensitivity toward Ganetespib could be confirmed in HCT116 cells containing a genetic deletion of the *USP22* gene (Supplementary Fig. S1D). Moreover, protein lysates isolated from *USP22* wild type and knockout cells were subjected to western blot analysis. In agreement with our hypothesis, Ganetespib-treated *USP22* knockout cells displayed increased induction of the apoptosis marker cleaved PARP (Supplementary Fig. S1E). In summary, these findings demonstrate that USP22-deficiency increases cellular sensitivity to HSP90i in vitro.

### *USP22*-deficient tumor cells display increased sensitivity to HSP90 inhibition

**Fig. 5:**
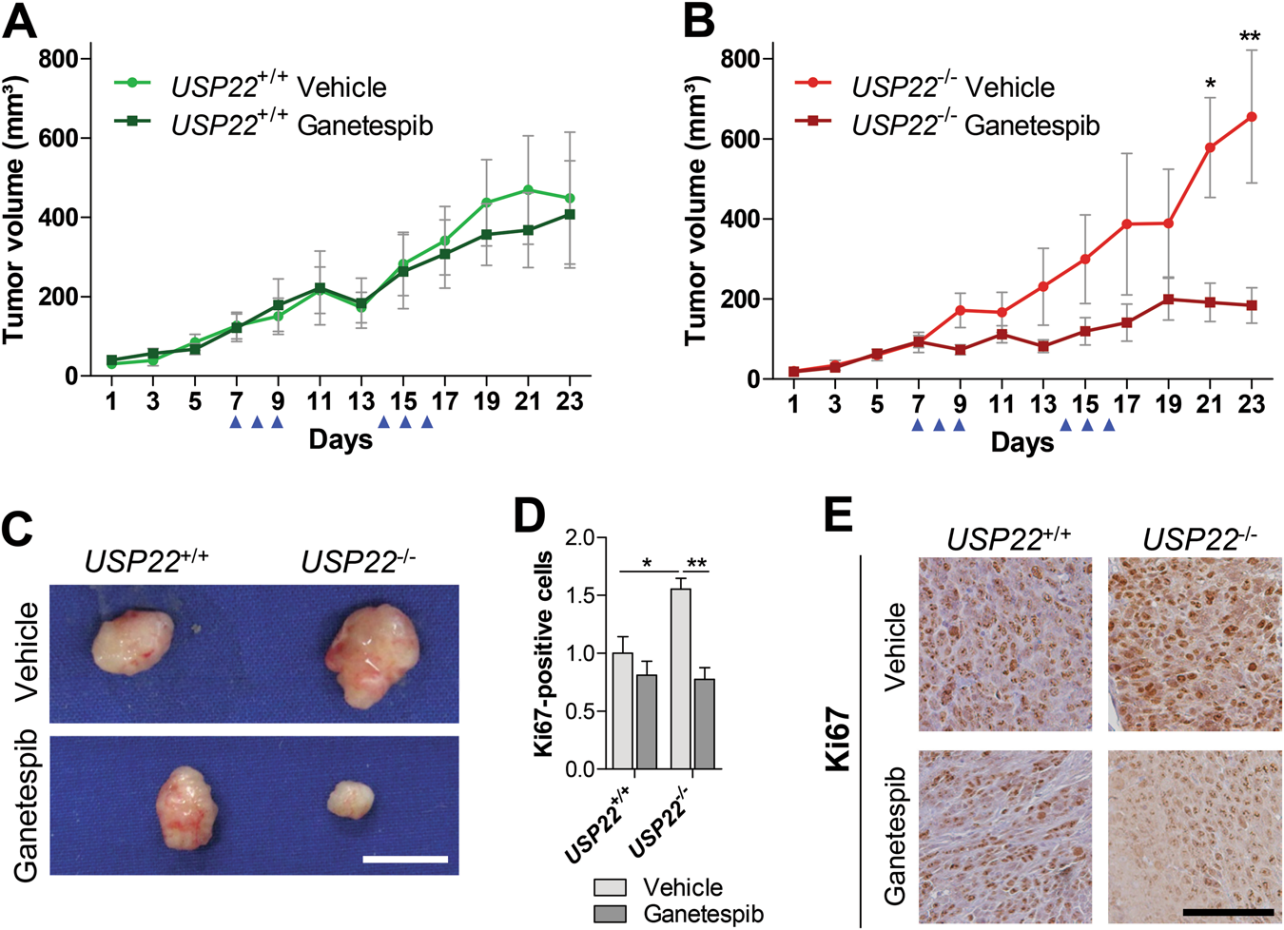
*USP22* deficiency conveys therapeutic vulnerability towards HSP90i in vivo. **a** In a xenograft approach, *USP22*^+/+^ and *USP22*^−/−^ HCT116 cells were injected subcutaneously into immunodeficient mice and treated with Ganetespib or vehicle (*n* = 6 per group). Results obtained from one representative wild type and *USP22* knockout clone are shown. While Ganetespib treatment displayed minor effects on *USP22* wild type tumors, **b** the accelerated growth of *USP22*^−/−^ tumors was significantly impaired by HSP90 inhibition to levels below that of treated *USP22*^+/+^ tumors. Blue arrow heads indicate the days on which Ganetespib was administered. Mean ± SEM, Student’s *t*-test. **c** Representative images of *USP22*^+/+^ and *USP22*^−/−^ tumors after the treatment with Ganetespib or vehicle. Scale bar: 1 cm. **d**, **e** As detected by IHC, the number of proliferating Ki67-positive cells in *USP22*^−/−^ tumors upon HSP90 inhibition was significantly decreased compared to tumors isolated from vehicle-treated mice. Mean ± SEM, one-way ANOVA. Scale bar: 100 µm.

Despite the fact that the majority of studies report oncogenic properties of USP22, we and others have discussed that USP22 might display tumor-suppressive functions in some biological contexts^6,26^. Thus, targeting USP22 could potentially have tumor-promoting effects. However, USP22-deficiency may introduce a tumor-specific therapeutic vulnerability that makes these tumors attractive targets for HSP90i treatment. To test this hypothesis in vivo, we utilized a xenograft-based approach to test the effect of Ganetespib treatment on *USP22*-deficient tumors in comparison to wildtype tumors. To this end, we injected *USP22*-deficient or wild type HCT116 cells subcutaneously into immunodeficient mice. Consistent with our previous results^6^, the loss of *USP22* accelerated HCT116 tumor growth. Importantly, intravenous injection of Ganetespib had a dramatic and preferential effect on *USP22* knockout cells where it had little effect on the growth of *USP22-*proficient tumors but significantly impaired the growth of *USP22-*deficient tumors more than 70% to a size 50% smaller than Ganetespib-treated wildtype tumors (Fig. 5a–c, Supplementary Fig. S1F). This finding was supported by immunohistochemical detection of the proliferation marker Ki67 (Fig. 5d, e). While the number of positively stained nuclei was increased after *USP22* loss, Ganetespib treatment significantly reduced proliferation and elevated apoptosis (Supplementary Fig. S1G, H) preferentially in *USP22*-deficient tumors. Taken together, our results suggest that *USP22* loss introduces a therapeutic vulnerability by sensitizing tumors towards HSP90 inhibition in vivo.

## Discussion

In the literature, USP22 was described to exert pro-tumorigenic functions in a wide variety of human malignancies and, therefore, USP22 would represent a promising therapeutic target. Thus, researchers are attempting to generate and optimize USP22 inhibitors^4^. In this study, we sought to build upon our previous results^6^ and identify common USP22-dependent molecular mechanisms in CRC and breast cancer. Intriguingly, we were able to demonstrate that HSP90AB1 levels are reduced upon USP22 loss in both colorectal and breast cancer cells in vitro. Interestingly, only the constitutively expressed HSP90β member HSP90AB1 was downregulated, while expression of the stress-inducible HSP90α isoforms were not affected.

Our findings support a direct role for USP22 as an essential component of the SAGA complex in promoting the expression of *HSP90AB1* during cancer development and progression. Increased expression of HSP90 family members has been associated with disease progression in the majority of human malignancies^27^ and HSP90AB1 has been described to play miscellaneous roles in human diseases due to the wide variety of client proteins^23^. Interestingly, while HSP90 is required for the stabilization of a number of oncogenes^28^, our findings suggest that the expression of its upstream regulator USP22 displays a dichotomous relationship with tumor progression where high *USP22* expression correlates with unfavorable prognosis in breast cancer but improved survival in rectal cancer patients. Indeed, this was reflected in our proliferation assays in which the siRNA-mediated knockdown reduced the confluence of HCC1954 cells and promoted growth of HCT116 cells. Surprisingly, but consistent with these findings, analysis of publicly available TCGA data published on the Human Protein Atlas website^22^ demonstrate that high *HSP90AB1* expression is also associated with poor prognosis in mammary carcinoma but a more favorable outcome in rectal carcinoma patients. Thus, decreased *HSP90AB1* expression in CRC with a poorer prognosis may represent an inadvertent bystander effect resulting from a CRC-specific selective pressure toward mTOR pathway activation as a consequence of *USP22* downregulation^6^. In agreement with the dichotomous tumor suppressive and promoting functions of USP22, a recent review questioned the reliability of several studies describing USP22 as an exclusively oncogenic factor due to the utilization of cross-reactive antibodies used for immunohistochemical stainings as well as the lack of appropriate controls^26^. These data indicate that, depending on the cancer type and the biological context, USP22 inhibition could either enhance or reduce tumor growth in patients. Thus, USP22 inhibitors which have been proposed to represent promising therapeutics might not display a suitable and safe treatment option in vivo.

Since HSP90 family members are involved in the cellular adaptation to stress^28^, we hypothesized that diminishing HSP90 completely would result in decreased survival of USP22-deficient cells. Indeed, we demonstrated that USP22 deficiency results in increased therapeutic vulnerability towards Ganetespib in vitro and in vivo. Notably, independent of the effects of USP22 loss on proliferation (decreased in HCC1954 and increased in HCT116 siUSP22 cells), a sensitization toward HSP90 inhibition was induced. Since various HSP90 inhibitors have been successfully tested in clinical trials^29^, targeting HSP90 may provide an ideal approach to exploit USP22-deficiency in cancer cells. Furthermore, combined inhibition of USP22 and HSP90 may provide an effective combinatorial approach to tumor therapy, which would overcome the context-dependency demonstrated in our studies. Interestingly, it was described that USP22 knockdown sensitized lung adenocarcinoma tumor spheres towards cisplatin treatment^30^. Similarly, Ganetespib-based HSP90 inhibition induced cell death via severe global chromosome fragmentation upon carboplatin treatment in vitro^31^. Future studies might reveal whether USP22 loss, and the subsequent reduction of HSP90 levels, will sensitize colorectal and breast cancer cells towards platinum-based therapy.

Together, in this study we demonstrate that the constitutively expressed heat shock factor *HSP90AB1* is highly dependent upon USP22-mediated epigenetic regulation in human breast and colorectal cancer cell lines as well as in murine mammary and colorectal tumors. Intriguingly, in a USP22-deficient context, the further inhibition of HSP90 activity using Ganetespib effectively reduced tumor growth in vitro and in vivo. Further investigations will reveal whether *USP22* expression levels can serve as a prognostic marker in cancer patients where low USP22 levels can be exploited by inducing therapeutic vulnerability to HSP90i treatment.

## Supplementary information

Supplementary Figure Legend

Supplementary Figure S1

Supplementary Tables
