# Supplementary material for: USP22-dependent HSP90AB1 expression promotes resistance to HSP90 inhibition in mammary and colorectal cancer
Source: Cell Death Dis. 2019 Dec 4;10(12):911. doi: 10.1038/s41419-019-2141-9 (PMC6892875; doi:10.1038/s41419-019-2141-9)
Supplement: Supplementary file 1 — Supplementary Figure Legend [file 41419_2019_2141_MOESM1_ESM.docx]

**Supplementary Figure S1: Sensitivity towards HSP90i in *USP22*^-/-^ HCT116 cells.** (A) Publically available gene expression data generated by the TCGA Research Network (<http://cancergenome.nih.gov/>;^22^) were analyzed for *USP22* expression in CRC and breast cancer (BC) patients. According to these data, a large proportion of colorectal cancer (22%; cut-off FPKM: 18.04) and breast cancer patients (26%; cut-off FPKM: 23.8) display low *USP22* expression. (B) The reduction of *HSP90AB1* mRNA levels was verified in HCT116 cells with a genetic *USP22* deletion (n=3). Mean ± SEM, *t*-test. (C) Knockdown efficiency of GCN5 in siRNA-transfected HCT116 cells (n=3). Mean ± SEM, *t*-test. (D) The sensitivity towards HSP90i was confirmed in two HCT116 wild type controls and two *USP22*^-/-^ clones after incubation with 100 nM Ganetespib for 48 h and crystal violet staining. (E) Treatment with 100 nM Ganetespib for 48 h increased the levels of cleaved PARP in *USP22*^-/-^ cells compared to wild type controls as demonstrated using western blot (n=3). (F) Xenograft tumor sizes of two HCT116 *USP22*^+/+^ and two *USP22*^-/-^ clones after treatment with Ganetespib or vehicle (n=6 per group). (G, H) As detected by IHC, the number of apoptotic cleaved caspase 3-positive cells upon Ganetespib treatment was increased in *USP22*^-/-^ tumors compared to wild type controls. Mean ± SEM, one-way ANOVA. Scale bar: 100 µm.
