# Supplementary figures and images for: USP22-dependent HSP90AB1 expression promotes resistance to HSP90 inhibition in mammary and colorectal cancer
Source: Cell Death Dis. 2019 Dec 4;10(12):911. doi: 10.1038/s41419-019-2141-9 (PMC6892875; doi:10.1038/s41419-019-2141-9)

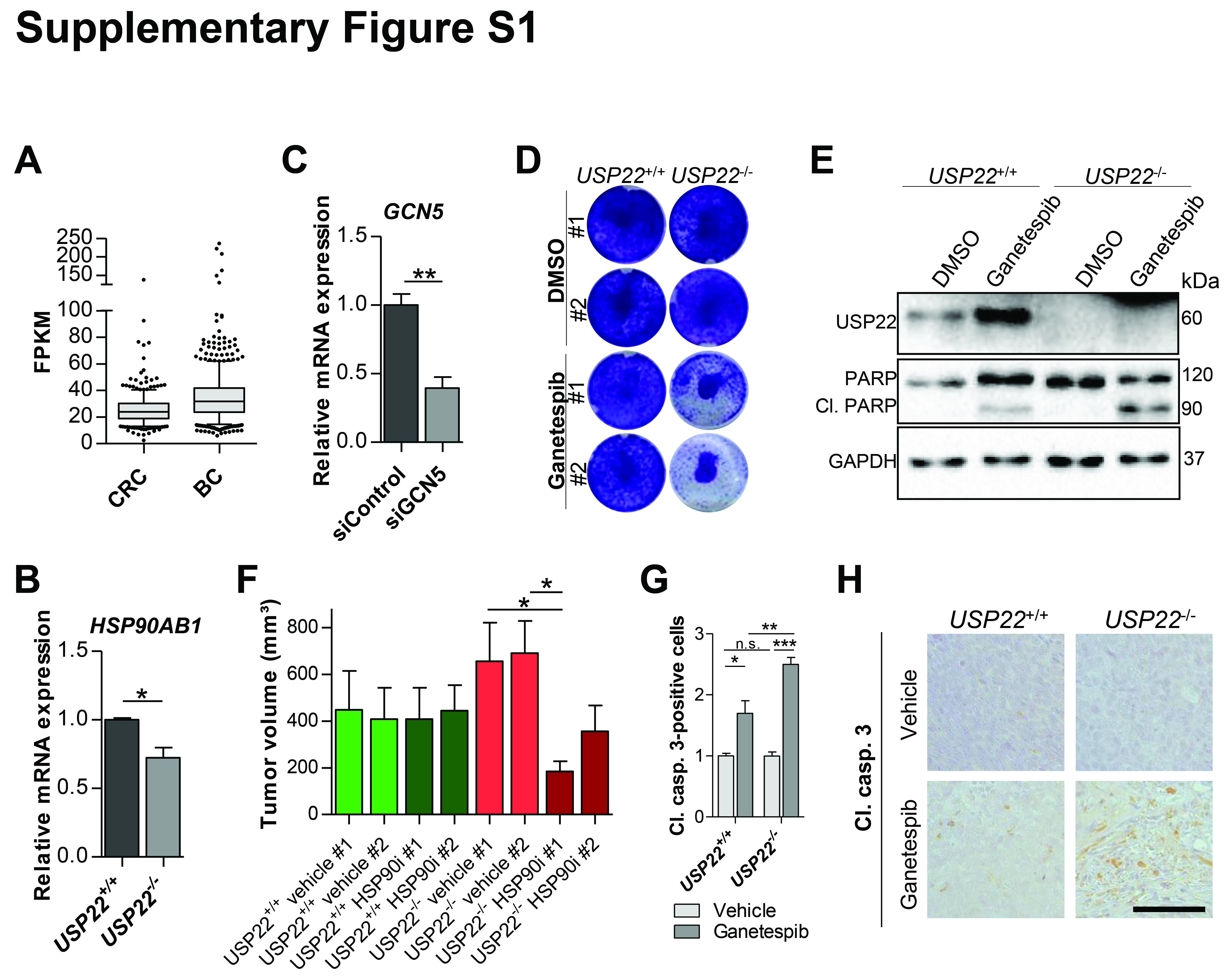

Supplement: Supplementary file 2 — Supplementary Figure S1 [file 41419_2019_2141_MOESM2_ESM.tif]
