# Supplementary material for: USP22-dependent HSP90AB1 expression promotes resistance to HSP90 inhibition in mammary and colorectal cancer
Source: Cell Death Dis. 2019 Dec 4;10(12):911. doi: 10.1038/s41419-019-2141-9 (PMC6892875; doi:10.1038/s41419-019-2141-9)
Supplement: Supplementary file 3 — Supplementary Tables [file 41419_2019_2141_MOESM3_ESM.docx]

**Table S1: Small interfering RNA.**

| **Gene** | **Cat. No.** | **Company** |
| --- | --- | --- |
| NT5 | D-001210-05 | Dharmacon |
| GCN5 | M-009722-01 | Dharmacon |
| USP22 | MU-006072-01 | Dharmacon |

**Table S2: Antibodies used for western blot and IHC.**

| **Antibody** | **Company** | **Cat. no** | **Western blot** | **IHC** |
| --- | --- | --- | --- | --- |
| Actin | Santa Cruz | sc-1616 | 1:5,000 |  |
| Cleaved caspase 3 | Cell Signaling | 9661 |  | 1:200 |
| GAPDH | Origene | TA802519 | 1:5,000 |  |
| H2B | Abcam | ab52484 | 1:20,000 |  |
| HSC70 | Santa Cruz | sc-7298 | 1:5,000 |  |
| HSP90AB1 | Millipore | AB3468 |  | 1:500 |
| HSP90AB1 (clone 4C10) | Origene | TA500494 | 1:5,000 |  |
| Ki67 | Abcam | ab115580 |  | 1:500 |
| USP22 | Santa Cruz | sc-390585 | 1:1,000 |  |

**Table S3: qRT-PCR primers.**

| **Gene** | **Sequence (5’-3’)** |
| --- | --- |
| *18SrRNA* | AACTGAGGCCATGATTAAGA |
|  | GGAACTACGACGGTATCTGA |
| *ABCE1* | TCTTTCGCCCAGTTATGGCA |
|  | TGCTATTTTGCTCTGGGGTGT |
| *CYCS* | GAGGCAAGCACAAGACTGGGCCAAA |
|  | TTATTGGCGGCTGTGTAAGAGTATC |
| *EIF4A1* | TATGACCTTCCCACCAACAGG |
|  | CAGCAACATTGAGGGGCATTT |
| *GCN5* | CATCGGTGGCATCTGCTT |
|  | GTACTCGTCGGCGTAGGTG |
| *HSP90AB1* | TTGACATCATCCCCAACCCTC |
|  | ACCAAACTGCCCAATCATGGA |
| *USP22* | AGCCAAGGGTGTTGGTCGCG |
|  | ACTGCCACCACGCCCGAAAG |

**Table S4: ChIP qRT-PCR primers.**

| **Primers** | **Sequence (5’-3’)** |
| --- | --- |
| *HSP90AB1* H3K9ac-positive | GGACGCGTTGTTAAAGCCTTC |
|  | GGAGACCGAAAGCTCTCTTCC |
| *HSP90AB1* H3K9ac-negative | AGAGAGCTAAGGCTCGTGGA |
|  | TCTTAAGCTCAGTGCCTGCC |
